# Egg Parasitoids of the Corn Leafhopper, *Dalbulus Maidis*, in the Southernmost Area of its Distribution Range

**DOI:** 10.1673/031.013.1001

**Published:** 2013-01-31

**Authors:** Eduardo G. Virla, Gustavo Moya-Raygoza, Erica Luft-Albarracin

**Affiliations:** 1 PROIMI-Biotecnología, Div. Control Biológico, CONICET, Av. Belgrano y Pje. Caseros (T400I MVB), San Miguel de Tucumán, Tucumán, Argentina; 2 Departamento de Botánica y Zoologáa, CUCBA, Universidad de Guadalajara, km 15.5 carretera Guadalajara-Nogales, Zapopan, C.P. 45110, Jalisco, México

**Keywords:** corn diseases, distribution edge, Mymaridae, natural enemies, Trichogrammatidae, vector

## Abstract

Egg parasitoids of the corn leafhopper, *Dalbulus maidis* (DeLong and Wolcott) (Hemiptera: Cicadellidae), were surveyed exposing sentinel eggs of the leafhopper along a latitudinal transect of 600 km in Argentina, the southernmost area of its distribution range. Four parasitoid species were obtained: the mymarids *Anagrus breviphragma* Soyka (Hymenoptera: Mymaridae), *Anagrus flaveolus* Waterhouse, and *Polynema* sp., and the trichogrammatid *Pseudoligosita longifrangiata* (Viggiani) (Hymenoptera: Trichogrammatidae). The low parasitism rate, low species richness, and high proportion of generalist egg parasitoids were quite clear in the southern distribution limit of the vector, in contrast to regions where corn crops are available all year round and there are continuous and overlapping generations of the pest. Further studies need to be done in order to determine the native host of the above egg parasitoids, the seasonal abundance, and the possible occurrence of other species affecting *D. maidis* populations in the studied area.

## Resumen

Los parasitoides de huevos de la chicharrita del maíz, *Dalbulus maidis* (DeLong and Wolcott), fueron estudiados exponiendo huevos trampas a lo largo de una transecta de 600 Km en Argentina, en su rango de distribución más austral. Cuatro especies de parasitoides fueron obtenidos: los mimaridos *Anagrus breviphragma* Soyka, *Anagrus flaveolus* Waterhouse, y *Polynema* sp., y el tricogrammatido *Pseudoligosita longifrangiata* (Viggiani). Una baja tasa de parasitoidismo, una baja riqueza de especies y una alta proporción de parasitoides de huevos generalistas, son bastante claros o evidentes en el limite de distribucion mas al sur de *D. maidis*, en contraste con las regiones donde el cultivo del maiz esta disponible durante todo el ano y las generaciones de la plaga son continuas y se superponen.

## Introduction

The corn leafhopper, *Dalbulus maidis* (DeLong and Wolcott) (Hemiptera: Cicadellidae), causes great damage to corn crops in most of the tropical and subtropical areas in the Americas because it efficiently transmits three important plant pathogens: Corn stunt spiroplasma (CSS), Maize bushy stunt phytoplasma (MBSP), and Maize rayado fino virus (MRFV) (Nault and Ammar 1989; Oliveira et al. 1998).

*D. maidis* shows a broad distribution throughout the Americas, having been found from southeastern and southwestern USA to Argentina (Triplehorn and Nault 1985). It only feeds on plants of the genus *Zea* (maize and teosintes) (Nault 1990).

In Argentina no host plant other than maize is available for the corn leafhopper. *D. maidis* is the most common leafhopper inhabiting corn crops north of parallel 30°, while further south its occurrence is sporadic (Paradell et al. 2001). Corn leafhopper populations reach their peak during the summer, but few adults survive the cold winter after senescence of maize plants starts and plants die off at the end of the growing season (Virla et al. 2003). Larsen et al. (1992), through greenhouse and laboratory experiments, and Moya-Raygoza et al. (2007), with field studies in Mexico, state that the vector is able to overwinter locally because it can survive at least two months in the absence of the maize host plants. Flight behavior studies conducted by Taylor et al. (1993) suggested that the corn leafhopper could travel long distances. In general, individuals disperse locally from areas with high-density populations (source populations) to unfavorable areas that are unable to support a viable population of the species throughout the year (sink population); entomologists have used the term “tourist” to describe these immigrant or non-resident species (Moran and Southwood 1982).

**Figure 1.**
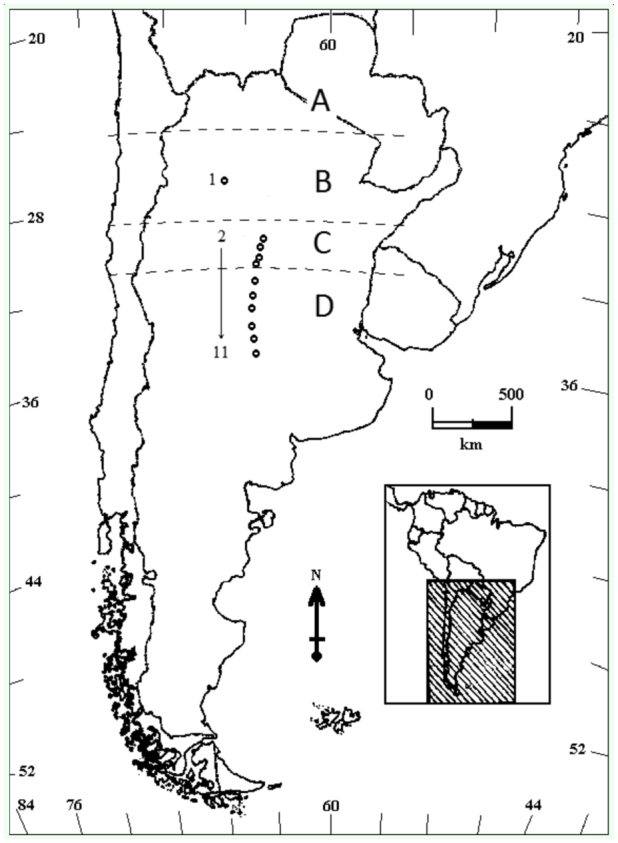
Distribution pattern of Dalbulus maidis in Argentina (A: tropical region, B: subtropical region, C: transition region, D: occasional region). Sample sites are labelled from I–II (for site references see Table 1). High quality figures are available online.

The distribution pattern of *D. maidis* in Argentina is mainly affected by climatic conditions that allow for its development and the availability of its host plants throughout the year. In general, there are four different regions along a latitudinal gradient in areas below elevations of 1,000 m.a.s.l. The “tropical region” (A in Figure 1) stretches from the border with Bolivia and Paraguay to approximately 24° S, just south of the tropic of Capricorn. In this region, corn crops are available all year round and there are continuous and overlapping generations of *D. maidis*. The “subtropical region” (B in Figure 1) is located between 24° and 28° S. In this region, adults overwinter using weeds and winter crops as refuge (Virla et al. 2003). The “transition region” (C in Figure 1) stretches from 28° to 31° S. In this region, vector populations develop during the summer and may survive year round, but only if winters are mild. The “occasional region” (D in Figure 1) is the area south of latitude 31°, where the presence of *D. maidis* populations is sporadic. Adults are only found at low densities, generally after midsummer, and they most likely do not survive winter (Virla, personal observation). In the subtropical and transition regions, tropical maize varieties are planted and corn crops are present between October and the beginning of May. In the occasional region, farmers sow temperate cultivars and the crops are present from September to the beginning of April. We suspect that in the occasional region, *D. maidis* populations derive from recolonization events that happen after unfavorable periods during winter, so they could be considered tourist species.

The corn leafhopper has a rich natural enemy complex. The egg parasitoids are well known in Argentina, where the vector is parasitized by 16 species. This knowledge is derived from studies carried out in tropical and subtropical areas of the country (Triapitsyn 1997; Luft-Albarracin et al. 2006; Luft-Albarracin and Triapitsyn 2007; Luft-Albarracin et al. in press; Polaszek and Luft-Albarracin 2011; Moya-Raygoza et al. 2012). The objective of the present study was to survey parasitoids that attack *D. maidis* eggs in the transition and occasional regions, which is the southern distribution limit of *D. maidis*.

## Materials and Methods

The *D. maidis* females used in this study came from a laboratory colony founded with individuals collected from corn crops in El Manantial (26° 50′ 03.41 S - 65° 16′ 30.62 W, 435 m.a.s.l.) (Tucumán province). The rearing of the insects was carried out in chambers under controlled conditions at 25 ± 3° C, 70–75% RH, and 14:10 L:D artificial photoperiod, using corn as the host plant.

Egg parasitoids were obtained by exposing sentinel eggs. In the laboratory, 6–10 female *D. maidis* were placed on maize leaves in cylindrical Polyethylene terephthalate (PET) cages (35 cm high × 18 cm diameter) to obtain eggs. Potted maize plants (6.3 dm^3^ pots) in the vegetative stage (3–6 leaves) were checked daily for eggs. The corn variety used was Leales 25 plus®. The number of sentinel eggs per potted plant was registered before field exposition.

Eggs less than 24 hrs old were exposed in cornfields for 72–96 hrs by placing them inside the corn field at not more than 3 m from the edge of the field. After eight days, the leaves containing exposed eggs were cut from the plant and transferred to a Petri dish containing wet tissue paper at the bottom, which was then covered with clear plastic wrap to avoid desiccation and to prevent wasps from escaping. Parasitized eggs were checked daily to ensure leaf quality until emergence of the adult wasps. The parasitoid specimens thus obtained were preserved in 70% ethanol and later slide-mounted in Canada balsam following traditional techniques. All emerging parasitoids were identified using specific keys (Triapitsyn 1997; Viggiani 1981). Voucher specimens were deposited in the entomological collection of the M. Lillo Institute, FMLA (Tucumán, Argentina).

Sentinel eggs were exposed from 16–20 March 2009 and 3–7 March 2010 along a transect of 600 km, mostly located between 29° and 35° S and 63° and 64° W (Figure 1). A total of 10 sample sites were selected (Table 1), in which the presence of nymphs and/or adults of the vector was checked by sweeping with a standard entomological net over the crop on the first and last day of the sentinel egg exposition. Each vector sample included a total of 50 sweeps per date.

**Table 1.**
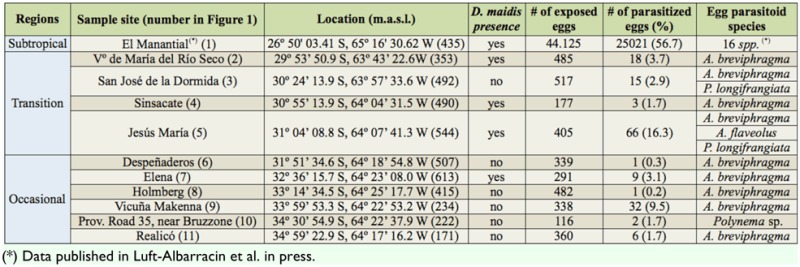
Sample sites in the transition and occasional regions of the southernmost distribution range of the corn leafhopper. Data include the total number of exposed sentinel eggs on maize crops, the number of parasitized eggs and the presence of the vector in the location. Data obtained at a site located in the subtropical area (1) are added as comparative information.

## Results

From a total of 3,510 exposed eggs, 153 (4.4%) were parasitized. At the sites located in the transition region, 1,584 eggs were exposed and 102 parasitized (6.4%). In the occasional region, 1,926 eggs were exposed and only 51 (2.6%) were attacked by egg parasitoids (Table 1).

If all parasitoids obtained along the transect are considered, four species affected the vector eggs: the mymarids *Anagrus breviphragma* Soyka (Hymenoptera: Mymaridae) (86.25% of the specimens), *Anagrus flaveolus* Waterhouse (2.5%), and *Polynema* sp. (2.5%), and the trichogrammatid *Pseudoligosita longifrangiata* (Viggiani) (Hymenoptera: Trichogrammatidae) (8.75%). The only species recovered from both the transition and occasional regions was *A. breviphragma*. The composition of the egg parasitoid complex in the northern sites (transition) was as follows: 80.4% *A. breviphragma*, 15.2% *P. longifrangiata*, and 4.4% *A. faveolus*. In the southern locations (occasional), where the presence of the vector is rare, two species were recovered: *A. breviphragma* (94.1%) and *Polynema* sp. (5.9%).

## Discussion

The mymarid wasp *A. breviphragma* attacks fourteen species (Triapitsyn 1997; Virla 2001; Luft-Albarracin et al. 2009), and although its broad host range is widely accepted, previous field studies (Virla 2001; Luft-Albarracin et al. in press) confirmed that it is hardly associated with *D. maidis* populations in Argentina. Interestingly, based on the available keys, the obtained *Polynema* sp. is possibly a new species, and previous attacks of the vector by this parasitoid have not been reported (Luft-Albarracin, personal observation).

The parasitism rates obtained in the current study are very low when compared with those obtained at “El Manantial” (subtropical region in the Tucumán province), where an average parasitism rate of 56.7% was obtained during a three-year study with exposed sentinel eggs using the same methodology (Luft-Albarracin et al. in press) (Table 1). Species richness was also low, as only three species were recovered from the transition region and two from the occasional area, compared with the species associated with the vector populations in the subtropical region. There are 16 egg parasitoids species affecting *D. maidis* populations in the subtropical region: *Encarsia dalbulae* Polaszek and LuftAlbarracin, *Aprostocetus* (O.) *infulatus* De Santis, *Anagrus breviphragma, A. flaveolus, A. miriamae* Triapitsyn and Virla, *A. nigriventris* Girault, *Polynema* sp. A, *Polynema* sp. B, *Aphelinoidea* sp. A, *Burksiella platensis* (De Santis), *Zagella nanula* De Santis, *Oligosita desantisi* Viggiani, *Oligosita giraulti* Crawford, *Pseudoligosita longifrangiata* (Viggiani), *Paracentrobia tapajosae* Viggiani and *Paracentrobia* sp. A (Luft-Albarracin et al. in press; Polaszek and Luft-Albarracin 2011; Moya-Raygoza et al. 2012).

Herbivore species colonizing new locations are often attacked by native parasitoid species. In these situations, overall parasitoid attack rates on invading hosts (a “tourist” species in this study) are generally lower than those on hosts normally developing in the region. Furthermore, parasitoid complexes on invading hosts are generally less rich and contain a higher proportion of generalists than those on native hosts (Cornell and Hawkins 1993; Vercher et al. 2005). The low parasitism rate, low species richness, and high proportion of generalist egg parasitoids was quite clear in the southern distribution limit of *D. maidis*.

Several authors (e.g., Huffaker et al. 1971; Huffaker and Messenger 1976; Waage and Greathead 1986; Dent 1991) have emphasized the need for knowledge of interrelationships of insect pests and their parasitoids in order to develop effective management tactics. In this context, the information given in this study could be useful for vector-control related programs.
